# Long-Term Neurodevelopmental Outcome of Monochorionic and Matched Dichorionic Twins

**DOI:** 10.1371/journal.pone.0006815

**Published:** 2009-08-28

**Authors:** Karien E. A. Hack, Corine Koopman-Esseboom, Jan B. Derks, Sjoerd G. Elias, Martin J. K. de Kleine, Wim Baerts, Attie T. J. I. Go, Arty H. P. Schaap, Mark A. H. B. M. van der Hoeven, Alex J. Eggink, Krystyna M. Sollie, Nynke Weisglas-Kuperus, Gerard H. A.Visser

**Affiliations:** 1 Department of Obstetrics, Wilhelmina Children's Hospital, University Medical Centre Utrecht, Utrecht, The Netherlands; 2 Department of Neonatology, Wilhelmina Children's Hospital, University Medical Centre Utrecht, Utrecht, The Netherlands; 3 Julius Centre for Health Sciences and Primary Care, University Medical Centre Utrecht, Utrecht, The Netherlands; 4 Department of Neonatology, Máxima Medical Centre, Veldhoven, The Netherlands; 5 Department of Neonatology, Isala Clinics, Zwolle, The Netherlands; 6 Department of Obstetrics, VU University Amsterdam, Amsterdam, The Netherlands; 7 Department of Obstetrics, Amsterdam Medical Centre, Amsterdam, The Netherlands; 8 Department of Neonatology, University Hospital Maastricht, Maastricht, The Netherlands; 9 Department of Obstetrics, Radboud University Nijmegen Medical Centre, Nijmegen, The Netherlands; 10 Department of Obstetrics, University Medical Centre Groningen, Groningen, The Netherlands; 11 Department of Neonatology, Erasmus Medical Centre, Rotterdam, The Netherlands; The University of Adelaide, Australia

## Abstract

**Background:**

Monochorionic (MC) twins are at increased risk for perinatal mortality and serious morbidity due to the presence of placental vascular anastomoses. Cerebral injury can be secondary to haemodynamic and hematological disorders during pregnancy (especially twin-to-twin transfusion syndrome (TTTS) or intrauterine co-twin death) or from postnatal injury associated with prematurity and low birth weight, common complications in twin pregnancies. We investigated neurodevelopmental outcome in MC and dichorionic (DC) twins at the age of two years.

**Methods:**

This was a prospective cohort study. Cerebral palsy (CP) was studied in 182 MC infants and 189 DC infants matched for weight and age at delivery, gender, ethnicity of the mother and study center. After losses to follow-up, 282 of the 366 infants without CP were available to be tested with the Griffiths Mental Developmental Scales at 22 months corrected age, all born between January 2005 and January 2006 in nine perinatal centers in The Netherlands. Due to phenotypic (un)alikeness in mono-or dizygosity, the principal investigator was not blinded to chorionic status; perinatal outcome, with exception of co-twin death, was not known to the examiner.

**Findings:**

Four out of 182 MC infants had CP (2.2%) - two of the four CP-cases were due to complications specific to MC twin pregnancies (TTTS and co-twin death) and the other two cases of CP were the result of cystic PVL after preterm birth - compared to one sibling of a DC twin (0.5%; OR 4.2, 95% CI 0.5–38.2) of unknown origin. Follow-up rate of neurodevelopmental outcome by Griffith's test was 76%. The majority of 2-year-old twins had normal developmental status. There were no significant differences between MC and DC twins. One MC infant (0.7%) had a developmental delay compared to 6 DC infants (4.2%; OR 0.2, 95% 0.0–1.4). Birth weight discordancy did not influence long-term outcome, though the smaller twin had slightly lower developmental scores than its larger co-twin.

**Conclusions:**

There were no significant differences in occurrence of cerebral palsy as well as neurodevelopmental outcome between MC and DC twins. Outcome of MC twins seems favourable in the absence of TTTS or co-twin death.

## Introduction

Monochorionic (MC) twins are at increased risk for perinatal mortality and serious morbidity compared to dichorionic (DC) twins [1]. The risk of cerebral palsy (CP) is increased among twins, which is mainly attributable to common complications in twin pregnancies such as preterm delivery and low birth weight [2]. However, several studies on birth weight specific CP prevalence have shown that the prevalence of CP is also higher in normal birth weight twins and is specifically associated with monochorionicity [2]–[4]. MC twin pregnancies complicated by intrauterine death of the co-twin or twin-twin transfusion syndrome (TTTS) carry a high risk of neurological impaired outcome due to cerebral ischaemia as a consequence of haemodynamic imbalance in the presence of placental vascular anastomoses [5]–[7]. However, even in the absence of either TTTS or single intrauterine fetal death, the occurrence of cranial ultrasound abnormalities and neuromorbidity is increased in (preterm) MC twins compared to DC twins [8]–[11]. Cerebral injury can be secondary to hemodynamic disorders during pregnancy or from postnatal injury associated with prematurity and low birth weight [9], [10], [12], [13].

Although long-term outcome has been studied extensively in TTTS [14]–[25] and co-twin death survivors [5], [6], [26], only a few studies investigated long-term outcome in MC and DC twin cohorts in general [8], [11]. In this prospective study, we therefore investigated long-term neurodevelopmental outcome of monochorionic and dichorionic twins at two years of age.

## Materials and Methods

All monochorionic (MC) twin pregnancies delivered between January 2005 and January 2006 in nine perinatal centers in The Netherlands, all tertiary referral centers with neonatal intensive care units (NICUs), were recorded. During the study period, 111 MC twin pairs were born in these hospitals. Eleven twin pregnancies were complicated by death of both infants. Four of the MC twins were monoamniotic; these pregnancies were excluded. Therefore, 96 MC twin pairs with at least one survivor were eligible for this prospective follow-up study. Perinatal mortality data of these twins will be reported separately in a large cohort, collected over a longer time period (Hack et al, submitted). A group of 98 dichorionic (DC) twins was matched for gestational age at delivery, gender, birth weight, ethnicity of the mother and study center.

The study was approved by the institutional review board of the University Medical Center Utrecht, and local permission was obtained in the other centers. Study participation by written consent was obtained from the parent(s). All twins with identical gender (i.e. boy-boy and girl-girl twins) were identified from the electronical databases of each hospital. We checked the medical charts to assess chorionicity. Chorionicity was determined on the basis of first-trimester ultrasound assessment and/or postpartum pathological examination of placentas and intertwin membranes. The diagnosis of TTTS was made antenatally by standard ultrasound criteria [27]. Twins were considered discordant in birth weight if the intertwin difference in birth weight, expressed as a percentage of the weight of the heaviest twin, was ≥20%. All twin gestations were antenatally monitored according to a standard protocol, which consisted of a first trimester determination of chorionicity, a detailed anomaly scan at 20 weeks of gestational age, and regular ultrasound assessment of growth, amniotic fluid volume and Doppler of the umbilical artery at least at 20, 24 and 28 weeks and fortnightly thereafter. Subjects with either nonreassuring fetal findings or with maternal complications were submitted to frequent but at least twice weekly maternal-fetal evaluations that were performed during hospitalization or during visits at an outpatient clinic setting. In seven out of 9 centers, elective delivery (i.e. induction of labour or caesarean section) of MC twins was offered around 37 weeks. In the other centers, MC pregnancy was only terminated in case of fetal and/or maternal complications.

Information on the occurrence of cerebral palsy (CP) was obtained from all children. The diagnosis of cerebral palsy (CP) was made according to standard criteria [28]. In case the developmental test could not be performed (due to lost-to-follow-up or non-participation) physical and neurological status was obtained from the attending pediatrician of the non-participants to be able to report the study population based incidence of CP. In the five cases where no follow-up data were available from a pediatrician, we assumed that children free of any complications related to CP, such as intraventricular hemorrhage (IVH) and periventricular leukomalacia (PVL), did not develop major neurological morbidity (i.e. CP).

The participants were tested with the Griffiths Mental Developmental Scales [29] by one single certified observer (KH) at a (corrected) age of 22 months and growth parameters (weight, height and head circumference) were measured. The investigator was first trained by a registered Griffith's course (Association for Research in Infant and Child Development, Southampton, UK) and examined a minimum of 20 infants under supervision of an experienced neonatologist working in a follow-up program before starting this study. The principal investigator was not blinded to chorionic status, since both infants might not look alike in case of dizygosity; perinatal outcome however, with exception of co-twin death, was not known to the examiner. Infants with CP were excluded, since some Griffiths subscales can not be reliably interpreted in case of motor disabilities. The diagnosis of CP was made by the attending paediatric neurologist, these children were not re-examined by the principal observer. The Griffiths Test investigates motor and mental development and detects possible delay or disability. A developmental quotient (DQ) of 100 is considered as the mean score. Mild developmental delay was defined as a DQ score between 68 and 84 (1–2 SD below the mean) and severe developmental delay was defined as a DQ of more than 2 SD below average (DQ≤68). Reported incidences of developmental delay (as defined above) and DQ-scores are based on the results of the Griffiths Test of the participating infants. The Health Status Classification System Preschool (HSCS-PS) questionnaire was completed by the parents before testing[30].

Statistical analysis was performed with the SPSS 12.0 statistical package. Continuous variables were compared by independent Student t-testing if normally distributed or otherwise by means of Mann Whitney U testing. Categorical variables were compared by Fisher's exact test. Linear regression was used to estimate mean differences of the DQ-scores according to chorionicity and donor or recipient of a TTTS-twin. These mean differences were adjusted for possible confounders. Statistical significance was based on two-sided tests with a cut-off level of 0.05.

## Results

**Figure 1:**
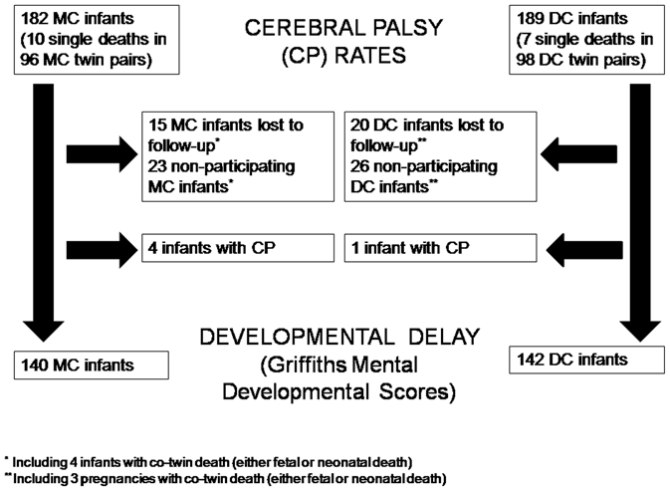
Flow diagram of study population.

During the study period 182 MC and 189 DC long-term survivors were approached for this prospective follow-up study. In forty-six twin pairs the developmental test could not be performed, either due to being lost-to-follow-up (15 MC and 20 DC infants) or parents did not approve participation (23 MC and 26 DC infants; Figure 1). Data on follow-up of 74 non-participating infants could be obtained from their pediatricians; this was restricted to CP only, and did not include development. Eight twin pairs did not have an attending pediatrician, since they were born at term and did not have any (developmental) problems. In 10 infants no follow-up data were retrieved; since none of these children had any major (neurological) complications during their neonatal period, we assumed that they did not develop CP. Characteristics of children who were tested by the Griffiths Test and non-participators did not substantially differ, except for a higher proportion of preterm born infants in the DC non-participators group.

### Cerebral Palsy (CP)

Four out of 182 long-term MC survivors (2.2%) and one out of 189 long-term DC survivors had CP (0.5%; OR 4.2, 95% CI 0.5–38.2). Two MC infants had CP due to complications specific to MC twin pregnancies. In one case fetal death of the smaller co-twin occurred at 26 weeks of gestation most likely due to placental insufficiency, with subsequent very preterm delivery of the surviving twin. This boy had a severe intraventricular hemorrhage (grade III) with posthemorrhagic ventricular dilatation and atrophy of brain tissue and developed spastic diplegia. In the other case, the pregnancy was complicated by TTTS (Quintero stage III) with laser coagulation of the connecting placental vascular anastomoses. Both infants were delivered at 37 weeks of gestation. The recipient showed normal development in contrast to the donor twin. The antenatal ultrasound of the brain showed echogenicity. The MRI after birth showed generalized cerebral atrophy and signs of an old hemorrhage in the periventricular area and in the basal ganglia. This child had a quadriplegia with severe mental retardation. The other two MC infants with CP were born very preterm (both at 28 weeks of gestational age with normal birth weight, two different pregnancies) and both developed spastic quadriplegia as a result of cystic periventricular leukomalacia (PVL). The cause of CP in the infant of the DC group (born at 31 weeks of gestation) is currently unknown, but may be genetically determined as this child also suffers from albinism. This child has diplegia with psychomotor retardation.

### Developmental status (results Griffiths Test)

After losses to follow-up, 140 MC infants and 142 DC infants without CP participated in this prospective follow-up study and were tested with the Griffiths Mental Developmental Scales. Baseline characteristics of the study population are shown in Table 1. There were significantly more female sex pairs among the DC twins. Seven pregnancies were complicated by single fetal demise (4 MC and 3 DC) and there were 3 neonatal deaths with survival of the sibling.

**Table 1:** Baseline characteristics and perinatal outcome of study population after losses to follow-up according to chorionicity.

Parameter	MC twins	DC twins
**No. of pregnancies studied**	73	73
**Socioeconomic status ‘highest’ parent, no. (%)**		
** low**	5 (6.5)	5 (6.8)
** middle**	21 (28.8)	22 (30.1)
** high**	47 (64.4)	46 (63.0)
**Ethnicity mother, no. (%)**		
** caucasian**	65 (89.0)	69 (94.5)
** mediterrean**	7 (9.1)	1 (1.4)
** asiatic**	0	1 (1.4)
** creole**	0	1 (1.4)
** hindustan**	1 (1.4)	1 (1.4)
**Fetal gender**		
** Female twin pairs, no. (%)**	35 (47.9)	44 (60.3)
**Pregnancies complicated by TTTS, no. (%)**	14 (19.2)	
**Pregnancies complicated by intra-uterine co-twin death, no. (%)**	4 (5.5)	3 (4.1)
**Perinatal outcome**		
**Infants born alive, no. (%)**	142	143
**Gestational age at delivery (wk), median and range**	35^+0^ (25^+2^–39^+6^)	35^+2^ (26^+6^–39^+3^)
**Birth weight of infants born alive (g),mean±SD**	2085±644	2131±600
**SGA (birth weight<p10), no. (%)**	23 (16.2)	35 (24.1)
Severe birth weight discordancy (≥20%) without TTTS	28 (24.6)	10 (13.7)
5-minute Apgar score, median (range)	9 (4–10)	9 (5–10)
**Umbilical artery pH, median (range)**	7.25 (7.00–7.48)	7.25 (6.86–7.44)
**Neonatal morbidity**		
** RDS, no. (%)**	24 (16.9)	16 (11.3)
** IVH, no. (%)**	8 (5.6)	5 (3.5)
** PVL, no. (%)**	0	0
** NEC, no. (%)**	2 (1.4)	0
**Neonatal death, no. (%)**	2 (1.3)	1 (0.7)
**Long-term survivors, no. (%)**	140	142

The majority of the tested infants showed normal development (98.2%). Five out of 282 long-term survivors had a mild developmental delay and two infants had a severe developmental delay. There was a significant difference in development between boys and girls. Boys had lower DQ scores (mean total DQ 101 and 106, respectively; p<0.001) and the proportion of mildly delayed development was higher among male infants. Infants born before 32 weeks of gestation and/or with a birth weight <1500 gram had significantly lower DQ-scores than infants born after 32 weeks and/or ≥1500 gram (mean total DQ 101 and 104, respectively; p = 0.05). There was no significant difference between infants who were small-for-gestational age and infants with a birth weight above the tenth percentile.

**Figure 2:**
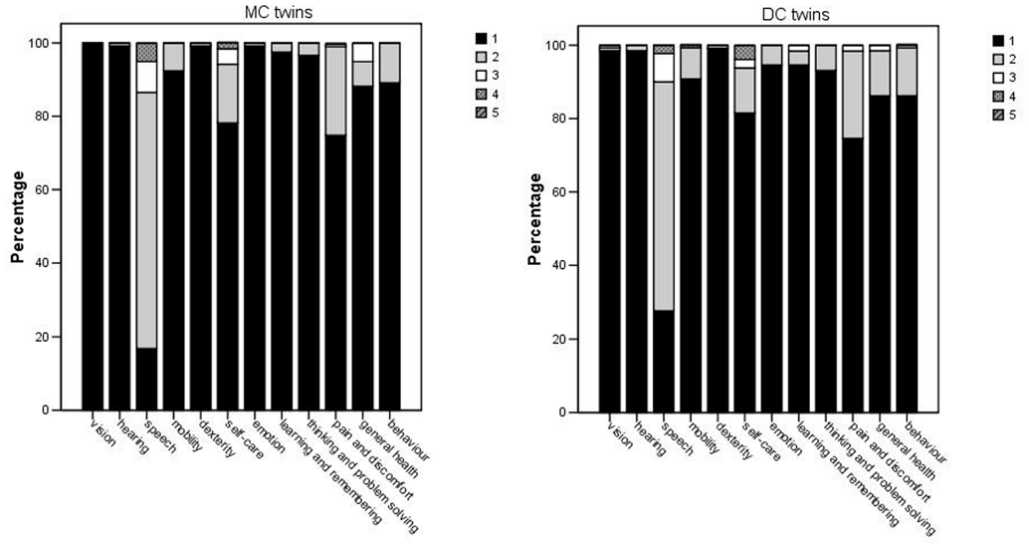
Health Status Classification System Preschool children (HSCS-PS) questionnaire completed by parents of monochorionic and dichorionic twins, each with three to five levels of severity.

Table 2 shows the long-term outcome of twins according to chorionicity. No differences in height, weight and head circumference between MC and DC twins were found. One MC infant (0.7%) had a developmental delay compared to 6 DC infants (4.2%; OR 0.2, 95% CI 0.0–1.4). When the population was divided in infants born before 32 weeks of gestation and/or with a birth weight <1500 gram and infants born after 32 weeks and/or a birth weight ≥1500 gram also no significant differences were present between the MC and DC twins (data not shown). Mean total DQ score was slightly lower in MC twins than in DC twins (mean TDQ 103 and 104, respectively, *p* = 0.423), which was mainly due to the higher proportion of mildly delayed hearing and speech development in MC twins. These results were in agreement with the parental impression of the development of their infant(s), as assessed by the HSCS-PS questionnaire (Figure 2).

**Table 2:** Outcome of twins at 2 years of age in relation to chorionicity.

Parameter	MC twins	DC twins	*P* value	Mean difference (95% CI)	Adjusted mean difference (95% CI)‡
**No. of long-term survivors**	140	142			
**Age at testing**					
Chronological age	23^+7^ months	23^+2^ months	0.178		
Corrected age	22^+3^ months	22^+2^ months	0.326		
**Physical exam**					
Height (cm), mean (SD)	86.0±3.4	86.5±3.7	0.239		
Weight (kg), mean (SD)	12.3±1.4	12.2±1.3	0.831		
Head circumference (cm), mean (SD)	48.6±1.6	48.6±1.4	0.978		
**Home environment**					
Older siblings	72 (49.0)	53 (37.3)	0.057		
multilingual education	17 (11.6)	9 (6.3)	0.151		
**Griffiths Test**					
**Total score** 1					
mean DQ (SD)	103±9	104±11	0.423	−0.96 (−3.33; 1.40)	0.34 (−1.88; 2.55)
Mild developmental delay	1 (0.7)	4 (2.8)	0.370		
Severe developmental delay	0	2 (1.4)	0.498		
**Subscale A; locomotor**					
mean DQ (SD)	93±10	94±13	0.493	−0.96 (−3.70; 1.78)	−0.48 (−3.26; 2.31)
Mild developmental delay	36 (26.5)	38 (26.8)	0.893		
Severe developmental delay	0	5 (3.5)	0.060		
**Subscale B; personal-social**					
mean DQ (SD)	103±10	102±12	0.593	0.69 (−1.87; 3.25)	1.93 (−0.54; 4.40)
Mild developmental delay	6 (4.8)	6 (4.2)	1.000		
Severe developmental delay	0	3 (2.1)	0.247		
**Subscale C; hearing & language**					
mean DQ (SD)	99±14	102±14	0.084	−2.82 (−6.01; 0.38)	−0.72 (−3.66; 2.23)
Mild developmental delay	19 (13.8)	8 (5.6)	0.026		
Severe developmental delay	1 (0.7)	5 (3.5)	0.214		
**Subscale D; eye-hand coordination**					
mean DQ (SD)	107±9	106±12	0.264	1.46 (−1.11; 4.03)	2.35 (−0.18; 4.88)
Mild developmental delay	1 (0.7)	4 (2.8)	0.371		
Severe developmental delay	0	2 (1.4)	0.492		
**Subscale E; performance**					
mean DQ (SD)	109±14	111±15	0.372	−1.56 (−5.00; 1.88)	−0.55 (−4.00; 2.90)
Mild developmental delay	5 (4.3)	5 (3.6)	1.000		
Severe developmental delay	3 (2.1)	2 (1.5)	0.683		

**Notes:**

1 In 10 cases incomplete testing (5 MC and 5 DC infants).

DQ = developmental quotient.

‡ Adjusted for gender, older sibling in family and multilingualism.

Developmental outcome of the twins after an uneventful course of pregnancy (i.e. the pregnancy was not complicated by preterm birth and/or a low birth weight <1500 g, TTTS or a birth weight discordancy of more than 20% or fetal death) is shown in Table 3. There were no differences between uncomplicated MC and DC twins, with exception of a significantly higher incidence of mildly delayed development of hearing and language in uncomplicated MC twins. Mean developmental score seems lower in uncomplicated MC twins compared to DC twins (103 vs 106, *p* = 0.109).

**Table 3:** Outcome of MC and DC twins after an uncomplicated course of pregnancy.

Parameter	uncomplicated MC infants	uncomplicated DC infants	P value	Mean difference (95% CI)	Adjusted mean difference (95% CI)^‡^
**No. of long-term survivors**	70	98	0.002		
**Physical exam**					
Height (cm), mean (SD)	86.4±3.3	87.0±3.5			
Head circumference (cm), mean (SD)	48.8±1.7	48.6±1.3			
Weight (kg), mean (SD)	12.6±1.5	12.4±1.2			
**Griffiths Test**					
**Total score**					
mean DQ (SD)	103±8	106±11	0.109	−2.53 (−5.62; 0.57)	−0.88 (−3.79; 2.04)
Mild developmental delay	0	0	-		
Severe developmental delay	0	2 (2.0)	0.512		
**Subscale A; locomotor**					
mean DQ (SD)	93±10	94±13	0.471	−1.34 (−5.00; 2.32)	−0.89 (−4.61; 2.85)
Mild developmental delay	18 (25.7)	26 (26.5)	1.000		
Severe developmental delay	0	2 (2.0)	0.511		
**Subscale B; personal-social**					
mean DQ (SD)	103±9	103±12	0.612	−0.82 (−4.12; 2.48)	1.00 (−2.25; 4.26)
Mild developmental delay	2 (2.9)	2 (2.0)	1.000		
Severe developmental delay	0	2 (2.0)	0.511		
**Subscale C; hearing & language**					
mean DQ (SD)	99±15	104±12	0.014	−5.24 (−9.38; −1.09)	−2.24 (−6.12; 1.64)
Mild developmental delay	12 (17.1)	3 (3.1)	0.002		
Severe developmental delay	1 (1.4)	2 (2.0)	1.000		
**Subscale D; eye-hand coordination**					
mean DQ (SD)	107±9	107±12	0.682	−0.69 (−4.16; 2.79)	0.63 (−2.86; 4.11)
Mild developmental delay	1 (1.5)	0	0.412		
Severe developmental delay	0	2 (2.0)	0.512		
**Subscale E; performance**					
mean DQ (SD)	110±13	112±14	0.225	−2.64 (−7.00; 1.71)	−1.61 (−6.00; 2.77)
Mild developmental delay	1 (1.5)	2 (2.0)	1.000		
Severe developmental delay	1 (1.5)	2 (2.0)	1.000		

Table 4 shows the outcome of discordant twins, which was also comparable between MC and DC twins. The smaller infant of the severely discordant twin pair had slightly lower DQ-scores compared to its larger sibling (mean TDQ 100 and 103, respectively; *p* = 0.338). This trend was found in both MC and DC discordant twins.

**Table 4:** Outcome of twins with a birth weight discordancy of at least 20% at 2 years of age in relation to chorionicity.

Parameter	Discordant MC twins pairs n = 14	Discordant DC twins pairs n = 10	P value	Mean difference (95% CI)	Adjusted mean difference (95% CI)‡
**No. of long-term survivors**	27	19			
**Physical exam**					
Height (cm), mean (SD)	84.3±2.8	84.0±3.8	0.768		
Head circumference (cm), mean (SD)	47.5±1.6	48.2±1.8	0.181		
Weight (kg), mean (SD)					
Smaller twin	10.9±0.9	11.0±1.6	0.841		
Larger twin	12.1±1.0	12.2±1.2	0.823		
Weight discordancy (%)	11.8±5.5	11.0±7	0.763		
**Griffiths Test**					
**Total score**					
mean DQ (SD)	102±10	99±13	0.338	−3.48 (−10.74; 3.78)	−1.61 (−9.00; 5.78)
Mild developmental delay	0	2 (11.8)	0.158		
Severe developmental delay	0	0	-		
**Subscale A; locomotor**					
mean DQ (SD)	91±9	89±15	0.698	−1.42 (−8.71; 5.88)	−2.12 (−10.42; 6.19)
Mild developmental delay	9 (33.3)	6 (31.6)	1.000		
Severe developmental delay	0	2 (10.5)	0.165		
**Subscale B; personal-social**					
mean DQ (SD)	102±12	100±11	0.686	0.12 (−6.84; 7.08)	3.30 (−4.02; 10.62)
Mild developmental delay	2 (7.4)	3 (15.8)	0.638		
Severe developmental delay	0	0			
**Subscale C; hearing & language**					
mean DQ (SD)	100±12	97±17	0.539	−2.72 (−11.57; 6.14)	−0.95 (−9.96; 8.05)
Mild developmental delay	3 (12.0)	2 (10.5)	1.000		
Severe developmental delay	0	2 (10.5)	0.181		
**Subscale D; eye-hand coordination**					
mean DQ (SD)	107±10	100±14	0.031	−7.96 (−15.14; −0.77)	−4.06 (−11.58; 3.47)
Mild developmental delay	0	3 (15.8)	0.064		
Severe developmental delay	0	0			
**Subscale E; performance**					
mean DQ (SD)	108±18	106±15	0.799	−1.33 (−12.08; 9.43)	−2.37 (−14.12; 9.38)
Mild developmental delay	2 (7.4)	1 (5.3)	1.000		
Severe developmental delay	1 (3.7)	0	1.000		

**Notes:**

DQ = developmental quotient.

‡ Adjusted for gender, older sibling in family and multilingualism.

Eighteen MC pregnancies were complicated by antenatal signs of TTTS (17.6%), of which six had been treated by laser coagulation of vascular anastomoses. Five pregnancies had been treated by serial amnioreduction. The other pregnancies did not receive any treatment, either due to low stage TTTS or immediate delivery after diagnosis. Three TTTS pregnancies were complicated by death of one of the twins. There were 33 long-term survivors, of which one had CP (3.0%). Twenty-seven long-term survivors were tested. All of them had a normal development (Table 5). However, recipients had slightly lower DQ-scores compared to the donor twins (mean TDQ 104 and 106, respectively; p = 0.640).

**Table 5:** Outcome of donor and recipient TTTS-twins at 2 years of age.

Parameter	Donors	Recipients	*P* value	Mean difference (95% CI)	Adjusted mean difference (95% CI)‡
**Birth weight (g)**	1675±621	1851±627	0.470		
**No. of long-term survivors**	14	14			
**Physical exam**					
Height (cm), mean (SD)	85.2±3.9	86.8±3.7	0.278		
Weight (kg), mean (SD)	12.0±1.2	12.7±1.4	0.171		
Head circumference (cm), mean (SD)	48.4±1.3	48.8±1.2	0.450		
**Griffiths Test**					
**Total score**					
mean DQ (SD)	106±6	104±9	0.640	−1.41 (−7.52; 4.70)	−0.75 (−6.23; 4.74)
Mild developmental delay	0	0			
Severe developmental delay	0	0			
**Subscale A; locomotor**					
mean DQ (SD)	96±9	95±12	0.784	−1.10 (−9.28; 7.09)	−1.17 (−9.99; 7.66)
Mild developmental delay	0	3 (21.4)	0.098		
Severe developmental delay	0	0			
**Subscale B; personal-social**					
mean DQ (SD)	108±8	105±10	0.468	−2.54 (−9.65; 4.56)	−1.80 (−8.66; 5.06)
Mild developmental delay	0	0			
Severe developmental delay	0	0			
**Subscale C; hearing & language**					
mean DQ (SD)	101±11	103±11	0.725	1.56 (−7.42; 10.53)	2.30 (−5.21; 9.81)
Mild developmental delay	1 (7.1)	1 (7.1)	1.000		
Severe developmental delay	0	0			
**Subscale D; eye-hand coordination**					
mean DQ (SD)	110±9	108±11	0.477	−2.68 (−10.31; 4.96)	−2.33 (−10.14; 5.48)
Mild developmental delay	0	0			
Severe developmental delay	0	0			
**Subscale E; performance**					
mean DQ (SD)	111±14	107±16	0.459	−4.36 (−16.29; 7.58)	−3.27 (−15.48; 8.95)
Mild developmental delay	1 (7.1)	1 (7.1)	1.000		
Severe developmental delay	0	0			

**Notes:**

Five TTTS pregnancies laser therapy.

DQ = developmental quotient.

‡ Adjusted for gender, older sibling in family and multilingualism.

In this cohort of 282 infants, four MC and three DC twin pregnancies were complicated by single intra-uterine death. In two MC cases IUD occurred after laser ablation for TTTS (at 18^+0^ and 25^+5^ weeks, respectively), one IUD at 26^+1^ weeks was most likely due to placental insufficiency, and the other IUD remained unexplained (at 32^+2^ weeks' gestation). One DC fetal death at 31^+1^ weeks was caused by placental insufficiency and the other two deaths remained unexplained (at 35^+4^ and 33^+6^ weeks, respectively). Only one (MC) survivor developed CP, all other survivors showed normal development.

## Discussion

Four MC infants had cerebral palsy (2.2%); two CP-cases were due to complications specific to MC twin pregnancies (TTTS and co-twin death, known risk factors for cerebral injury in the surviving MC twin) and two cases of CP as a result of cystic PVL after preterm birth. One DC twin had CP (0.5%) without clear cause. The majority of 2-year-old twins had a normal developmental status. There were no significant differences between MC and DC twins, apart from a slight delay in hearing and language development in MC twins. Birth weight discordancy did not influence long-term outcome, although there was a trend towards lower DQ-scores in the smaller twin than in its larger co-twin.

The response rate of this follow-up study was 76%. This raises concern about the possibility of a non-response bias. In order to reduce this possibility to a minimum, we obtained information on neurological status and development from the attending pediatrician of the non-participants. In this way, we were able to report the study population based CP incidence. From five twin pairs no follow-up data were retrieved. We made an assumption that these children did not develop CP, since they did not have any major (neurological) complications during their neonatal period.

A limitation of this study is that it was not population-based. The data in this study should thus be interpreted with care since a selection bias may have been introduced due to the specific nature of tertiary referral centres. However, long-term outcome of the twins was even better than reported in previous publications.

With regard to the analyses based on the Griffiths test, it may be that our results are somewhat biased in favor of the DC group. That is, non-responders in the DC group were found to be born earlier than the responders in this group, a difference that was not found in the MC group, which may have led to an underestimation of developmental delay especially in the tested DC group. However, since we expected a worse outcome of MC twins due to possible cerebral damage in the presence of haemodynamic imbalance, this bias does not influence our conclusion that long-term outcome of MC twins was not shown to be worse than that of DC twins.

However, it should be noted that neurological handicaps and mental retardation may become evident only several years after birth, so a new evaluation of CP and developmental delay at the age of five years is preferable.

It is well known that the incidence of abnormal long-term neurodevelopmental outcome in MC pregnancies complicated by TTTS is high. After treatment with serial amniodrainage, most studies on long-term outcome report a 22–26% incidence of neurological impairment [14]–[17], [19], [20]. Neurodevelopmental outcome of TTTS-survivors has improved considerably with the introduction of laser coagulation of the placental vascular anastomoses with reported incidences of 6–17% [12], [21]–[25], [31]. We found a low incidence of neurodevelopmental delay in our TTTS-group. Possibly, the more complex TTTS pregnancies with adverse long-term outcome were delivered in the Leiden University Medical Center, the national referral center for TTTS in The Netherlands. Their data were not included in our series, and their follow-up data have been published separately [12], [14], [25]. They found a 10% incidence of antenatally acquired cerebral lesions in the TTTS-group and 2% in the non-TTTS group [12]. The incidence of CP was high (21%) in TTTS infants treated by serial amniodrainage or managed conservatively [14], whereas 7% of TTTS pregnancies treated by fetoscopic laser surgery had CP (7%); 7.8% had a mental developmental delay and 10.4% of the infants had a psychomotor developmental delay at the age of two years. Although we found a slightly lower DQ-score in the recipient twin, we did not find significant differences in long-term outcome between donor and recipient twins, which is in agreement with the data from Lopriore and others [14], [23]–[25]. However, we report a rather low incidence of CP among TTTS-survivors (3.0%) compared to other studies (5 to 9%) [18], [19], [22], [25].

It is also well known, that the risk of neurological abnormality in the surviving twin after co-twin intra-uterine death is higher in MC than in DC twins. The mechanism leading to damage of the surviving sibling is an acute blood loss through the placental vascular anastomoses into the dying fetus, leading to hypovolemia in the survivor, which in turn might cause death from hypovolemic shock or cerebral ischemia due to hypoperfusion [32].We found that one out of four surviving MC siblings had CP compared to none of the surviving DC infants. This is in agreement with previous studies [5], [6].

Intrauterine growth restriction (IUGR) is another important risk factor for cerebral palsy and impaired neurological development. Gratacos and co-workers [33] found that selective IUGR in MC twins is associated with an elevated risk of intra-uterine demise (IUD) of the smaller twin and neurological damage in the larger twin (the latter not restricted to cases with IUD of the co-twin). However, we did not find any major differences between the smaller and the larger twin in the subgroup of severe birth weight discordant twins (as a reflection of selective IUGR), and even found the smaller twin to perform slightly worse than its larger sibling, which is in agreement with previous studies [34], [35].

The increased risk of neurological disability in MC survivors of TTTS or co-twin fetal death suggests that cerebral injury in MC twins is related to the vascular anatomy of the placenta. Placental vascular anastomoses, which are nearly always present, can cause haemodynamic imbalance and subsequent ischemic injury to the brain. Cranial ultrasound abnormalities in the neonatal period also suggest that monochorionity itself is a risk factor for cerebral damage [9], [10], [36]. Although long-term outcome has been studied extensively in TTTS and co-twin death survivors, only two studies have been published on long-term outcome in MC and DC twins in general. In a Japanese study [11], infant outcome at one year of age was studied in 44 MC pregnancies and 164 DC pregnancies (study period 1990–1996). Adverse outcome, including CP and mental retardation (the latter diagnosis being based on the results of a Japanese developmental test), occurred in 9 out of 88 MC infants (10.2%) and in 12 out of 328 DC infants (3.7%, *p*<0.05). TTTS was considered to be responsible for most cases of adverse outcome in the MC group. In the same period, Adegbite and collegues [8] studied 76 MC and 78 DC twins (295 infants), born preterm between 24 and 34 weeks of gestation. They found a higher incidence of CP (8% and 1%, respectively) and neurologic morbidity (15% and 3%, respectively) in MC twins compared to DC twins. The risk of impaired neurodevelopment was specifically high in the presence of birth weight discordancy, TTTS and co-twin death. We found considerably lower incidences of neuromorbidity without significant differences between MC and DC twins compared to the reported long-term outcome of the mid 1990s. In contrast to these previous studies, we did not study a general population of DC twins, but we matched the DC twins for birth weight and gestational age at delivery. MC twins generally deliver earlier and have lower birth weights, which are both factors associated with impaired long-term neurodevelopmental outcome. Moreover, current obstetric management of the MC twin, which has improved considerably during the past decade, has ameliorated perinatal survival and neonatal outcome. In our study group impaired neurodevelopmental outcome was only due to complications specific to MC twin pregnancies (TTTS and co-twin death) or due to preterm delivery. Recently, Ortibus et al prospectively studied a large cohort of MC diamniotic twins, in which the CP-rate was 2% and impaired neurodevelopmental outcome occurred in 7.4% of 2-year-old infants. They found one infant with CP after an uneventful pregnancy and term birth [37].

In summary, the majority of 2-year-old twins had a normal neurodevelopmental status. There were no significant differences between MC and DC twins. CP may occur more frequently in MC twins and was mainly due to complications specific to MC twin pregnancies (TTTS and co-twin death). Outcome of MC twins was favourable in the absence of TTTS or co-twin death. More studies are needed to evaluate the effect of improved obstetric management on the long-term neurodevelopmental status of ‘uncomplicated’ MC twins. Meanwhile we suggest a routine cranial ultrasound examination after birth and neurodevelopmental follow-up.
